# Laparoscopic-enhanced recovery after surgery protocol for incarcerated inguinal hernia: a paradigm shift toward precision emergency herniology

**DOI:** 10.3389/fsurg.2025.1626717

**Published:** 2025-08-13

**Authors:** Xinqiang Zhu, Siwei Shan, Jianwei Zhang

**Affiliations:** ^1^ Department of General Surgery, The Affiliated Suqian Hospital of Xuzhou Medical University, Suqian, Jiangsu, China; ^2^ Department of Ultrasound, The Affiliated Suqian Hospital of Xuzhou Medical University, Suqian, Jiangsu, China

**Keywords:** laparoscopic, enhanced recovery after surgery, hernia, inguinal, incarcerated

## Abstract

**Objective:**

To evaluate the safety and efficacy of a modified Enhanced Recovery After Surgery (ERAS) protocol integrated with laparoscopic repair for incarcerated inguinal hernia, comparing outcomes with conventional open surgery.

**Methods:**

This single-center retrospective cohort study (2019-2024) included 200 patients with incarcerated inguinal hernia. These patients were assigned to the laparoscopy group or the open group (in a 1:1 ratio). Propensity score matching (PSM) balanced the baseline characteristics. ERAS intervention includes preoperative counseling, multimodal analgesia and forced early activities. Continuous variable: Independent *t*-test or Mann–Whitney U; Categorical variables: Chi-square test or fish test; Multivariate logistic regression was used for hazard ratio analysis.

**Results:**

After PSM (80 pairs), the laparoscopic group demonstrated significantly lower overall complications (9% vs. 38%, *P* = 0.007), including reduced surgical site infections (6% vs. 18%) and postoperative ileus (4% vs. 14%). Laparoscopy shortened hospital stays (3.1 vs. 5.6 days, *P* < 0.001), accelerated bowel function recovery (16.5 vs. 26.3 h, *P* < 0.001), and decreased opioid use (12.4 vs. 32.7 mg, *P* < 0.001). Eighteen cases required open conversion (15 for intestinal resection). No large bowel resections occurred.

**Conclusion:**

Laparoscopic repair of incarcerated inguinal hernias integrated with ERAS protocols demonstrates significant clinical efficacy, effectively reducing postoperative complications and accelerating recovery, thereby establishing itself as a recommended standard for widespread clinical adoption.

## Introduction

Incarcerated inguinal hernia is a life-threatening surgical emergency, and studies have shown that emergency patients account for about 25% of all inguinal hernias (1, 2). Immediate surgical intervention is mandatory to prevent intestinal strangulation and systemic sepsis. However, this operation has obvious risks, especially for elderly patients with cardiopulmonary complications, and the postoperative mortality can reach about 5%, which is 7 times higher than that of elective surgery (3). While traditional open approaches remain effective for rapid decompression, they are associated with prolonged recovery and increased wound-related morbidity (4). Enhanced Recovery After Surgery (ERAS) protocols have demonstrated remarkable success in elective hernia repair, reducing hospital stays and opioid consumption through multimodal analgesia, early mobilization, and standardized perioperative care (5). Despite this progress, the application of ERAS principles to incarcerated inguinal hernia remains underexplored and inconsistently implemented in clinical practice. Although preliminary exploratory studies suggest that ERAS may be safe and effective in rigorously selected cases of incarcerated hernia (6), no consensus exists regarding protocol adaptation for this high-risk population. We propose a modified ERAS pathway integrating intraoperative bowel viability assessment and risk-stratified postoperative management. This retrospective cohort study aims to evaluate whether this optimized protocol can achieve comparable safety profiles to conventional care while accelerating functional recovery in patients with incarcerated inguinal hernia.

## Materials and methods

### Patients and procedures

The inclusion criteria for patients were as follows: (1) adult inguinal hernia, age ≥18 years old. (2) No history of abdominal surgery and (3) The vital signs were stable and there was no septic shock.

Exclusion criterion for patients were as follows: (1) cardiopulmonary insufficiency, unable to tolerate anesthesia. (2) History of abdominal surgery. (3) Confirm necrosis and infection of hernia contents and (4) Pregnancy, immunodeficiency.

This was a single-center, retrospective cohort study conducted at Suqian Hospital Affiliated to Xuzhou Medical University from 2019 to 2024. Patients meeting predefined eligibility criteria for incarcerated inguinal hernia were prospectively assigned to undergo either laparoscopic or open surgical repair in a 1:1 allocation ratio. To minimize selection bias, propensity score matching (PSM (7, 8) was performed using variables including age, sex, BMI, ASA class, Bowel ischemia.

### Ethics and consent

The study was conducted in accordance with the Declaration of Helsinki, and signed informed consent was obtained from the patients. The study has been approved by the Ethics Committee and institutional review board of Suqian Hospital Affiliated to Xuzhou Medical University.

### Treatment

All patients were given tracheal intubation anesthesia and ultrasound-guided transversal planar nerve block (TAP). Endoscopic group: release and reduction of incarcerated hernia through abdomen, repair without preperitoneal tension. In the open group, longitudinal incision on the surface of the mass was selected for exploration, incision of the hernia sac, exploration of the hernia contents, and release of the hernia ring. Select the mesh plug for repair. Both groups were repaired with domestic Shan-release patch, and both groups were operated by the same group of doctors.

### ERAS protocol implementation

Both groups received ERAS intervention unless contraindicated (in the case of enterectomy, early eating is not condoned). Key components included: preoperative communication, multimodal analgesia (TAP block + NSAIDs), and forced activity within 6 h after surgery.

### PSM details

To address selection bias in this observational, propensity scores were generated using binary logistic regression in SPSS.1:1 nearest-neighbor matching was performed with a caliper of 0.2 SD using the *PS Matching* SPSS19 extension. Balance was verified by standardized mean differences.

### Statistical methods

The statistical analysis was conducted using SPSS 19.0 software. The categorical data are presented as percentages, and the description of continuous data can be achieved by calculating standard deviations or employing medians. Continuous variables: Independent *t*-test or Mann–Whitney U; Categorical variables: Chi-square or Fisher's exact test; Multivariate logistic regression was used for hazard ratio analysis. The test level was *α* = 0.05.

## Results

A total of 200 patients were included in the final analysis, with 100 allocated to each group. After propensity score matching, 80 pairs were well-balanced in baseline characteristics. No significant differences remained in age, male (%), ASA III-IV (%), BMI, or bowel ischemia (*P* > 0.05) (Table 1).

**Table 1:** Baseline characteristics before and after PSM.

Variable	Before PSM		After PSM	
	Laparoscopic	Open	Laparoscopic	Open
Age (years)	65.2 ± 10.1	68.3 ± 9.5*	66.0 ± 9.8	66.4 ± 10.2
ASA III-IV (%)	22%	30%	25%	24%
Male (%)	94%	87%	90%	90%
BMI ≥30 (%)	32%	26%	30%	31%
Bowel ischemia	10%	16%	13%	14%

* *P* < 0.05 before matching; all *P* > 0.05 after matching.

**Figure 1:**
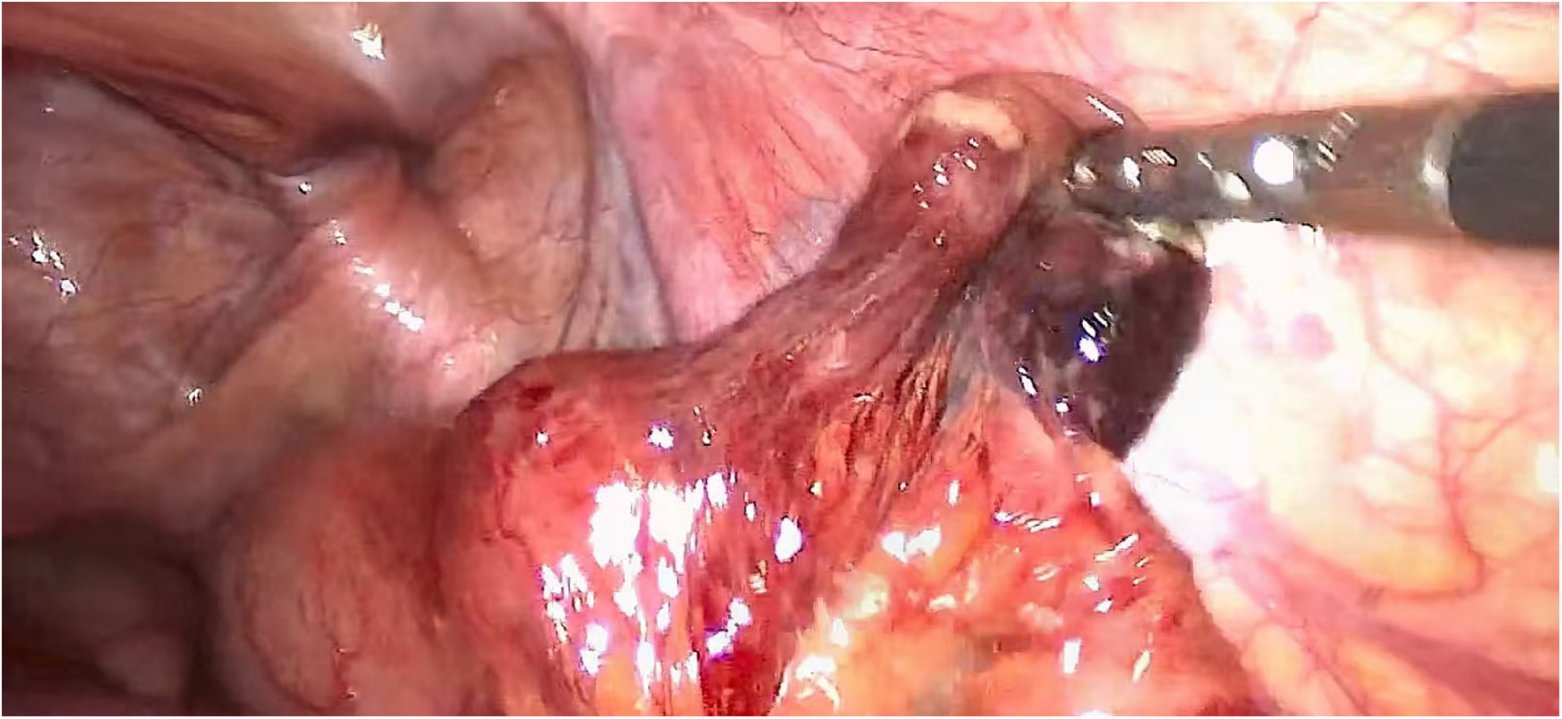
Incarcerated intestinal tubes, after release, the vitality of the intestinal tubes was observed, and there was no necrosis. It enables a more comprehensive observation of the incarcerated intestinal tract.

**Figure 2:**
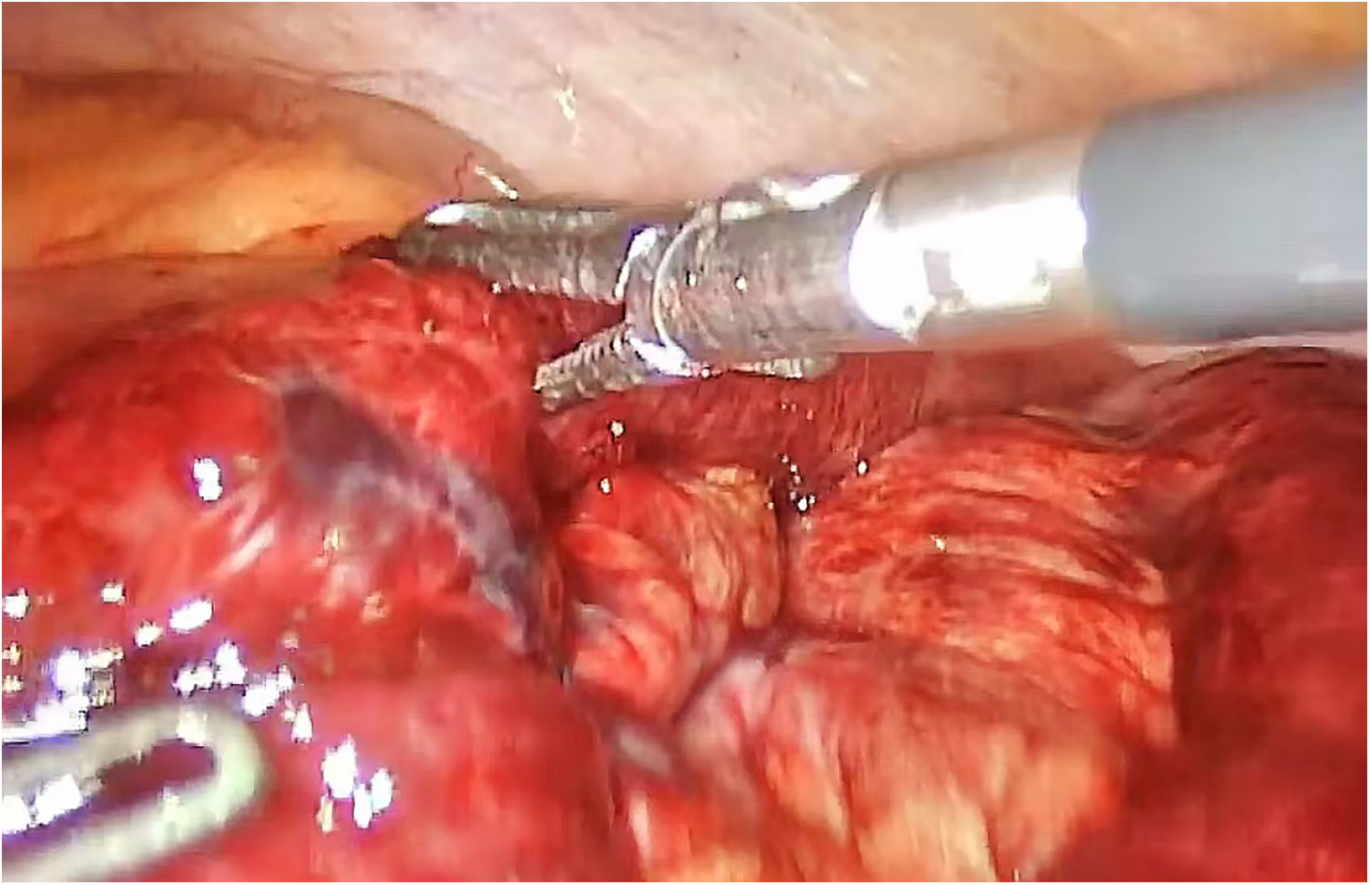
Incarceration is a part of the intestinal wall with local ischemic necrosis, and resection and anastomosis must be performed.

Overall complication details were reduced in the laparoscopic group compared with the open group (9% vs. 38%, *P* = 0.007), mainly due to lower rates of surgical site infection (6% vs. 18%) and intestinal obstruction (4% vs. 14%) (Table 2). The intestinal duct activity can be comprehensively observed under endoscope. After the preperitoneal tension-free repair is completed, there is sufficient time to observe intestinal viability. Bowel color, bowel peristalsis capacity, and peripheral blood vessel pulsation were mainly observed (Figure 1). Of course, there are also intestinal tubes with poor vitality that need to be surgically removed. In this study, 15 patients underwent laparotomy (Figure 2), and 3 patients with incarcerated hernias were unable to be reduced under endoscopy and underwent assisted open surgery.Patients undergoing laparoscopic repair had significantly shorter hospital stays (3.1 ± 1.2 vs. 5.6 ± 2.4 days, *P* < 0.001), earlier return of bowel function (16.5 ± 5.8 vs26.3 ± 8.9 h, *P* < 0.001), and reduced opioid consumption (12.4 ± 4.2 vs32.7 ± 10.5 mg, *P* < 0.001) (Table 3).

**Table 2:** Comparative analysis of perioperative outcomes between laparoscopic and open groups (primary outcomes).

Variable	Laparoscopic group (*n* = 80)	Open surgery group (*n* = 80)	Risk ratio (95% CI)	*p*-value
Overall complications*	9%	38%	0.55 (0.37–0.83)	0.007
Surgical site infection	6%	18%	0.33 (0.14–0.78)	0.01
Postoperative ileus	4%	14%	0.29 (0.11–0.74)	0.02
Enterectomy anastomosis	15%	23%	0.46 (0.29–1.44)	0.26
Open transfer operation	18%	0	0.39 (0.21–0.88)	0.03
Cardiopulmonary events	5%	9%	0.56 (0.23–1.36)	0.2
DVT/PE	2%	3%	0.67 (0.15–2.95)	0.65

*Overall complications include surgical site infection, postoperative ileus.

**Table 3:** Comparative analysis of perioperative outcomes between laparoscopic and open groups (secondary outcomes).

Variable	Laparoscopic group (*n* = 80)	Open surgery group (*n* = 80)	Risk ratio (95% CI)	*p*-value
Length of stay (days)	3.1 ± 1.2	5.6 ± 2.4	–	<0.001
VAS pain score (24 h)	2.8 ± 1.1	4.5 ± 1.3	–	<0.001
Time to flatus (hours)	16.5 ± 5.8	26.3 ± 8.9	–	<0.001
Opioid use (OME, mg)	12.4 ± 4.2	32.7 ± 10.5	–	<0.001
90-day readmission rate	3%	5%	0.63 (0.25–1.56)	0.41

## Discussion

The present study challenges the conventional paradigm of open surgery as the default approach for incarcerated inguinal hernia by demonstrating that laparoscopic repair, when integrated with ERAS principles, significantly reduces complications and accelerates recovery without compromising safety. Our findings extend the frontier of minimally invasive emergency surgery and warrant a critical reappraisal of current clinical guidelines.

1. ERAS in Emergency Hernia Surgery: Safety, Efficacy, and Accelerated Recovery Through Minimally Invasive Approaches

Enhanced recovery after surgery protocols have revolutionized elective hernia treatment (9, 10), but their use in the emergency department has been limited due to concerns about bowel motility and hemodynamic instability. The waiting time can be directly observed during the operation, which is easier to judge the tissue activity, reduce complications, and is more conducive to the early implementation of ERAS after surgery. Importantly, our protocol implemented ERAS early in all cases, resulting in rapid recovery and without excessive complications, providing a rationale for the safety of ERAS applied to incarcerated hernias. The application of ERAS in hernia and abdominal wall surgery has been implemented. The introduction of ERAS pathway may reduce the length of hospital stay、pain and trauma of patients with abdominal wall reconstruction (11). The minimally invasive advantages of laparoscopy, more accurate dissection (12), make rapid recovery easier to implement.

2. Advantages of laparoscopy in the treatment of incarcerated inguinal hernia

Despite conventional wisdom that laparoscopic surgery increases the risk of intestinal damage, our data showed that the laparoscopic group had a lower incidence of intestinal obstruction (4% vs. 14%) and a faster rate of intestinal recovery. Precise dissection under enlarged field of view can noninvasively reduce the edema of the bowel (13). It is more beneficial to observe the activity of impacted tissue and conduct more comprehensive exploration of impacted tissue such as bowel duct and omentum (14). At the same time, the pressure of pneumoperitoneum can resist the force of the abdominal wall muscle tissue, which can better release the hernia ring and facilitate the reduction. In this study, 18 cases were transferred to open surgery, 15 of which required small intestine resection and anastomosis. In the other 3 cases, it was necessary to jointly open and relax the herniation ring, and incarcerate the intestinal tube. Some studies have shown (15) that emergency small intestine resection combined with patch repair is a safe treatment. Of course, it did not include large intestine resection, and in our study, there were no cases of large intestine resection. Endoscopic surgery can reduce the degree of tissue injury in open surgery and improve the surgical comfort of patients. Laparoscopic minimally invasive surgery can significantly reduce the trauma of open surgery, alleviate pain, and facilitate rapid recovery. The safety of laparoscopic treatment has been recognized (16), and in 2013, the European Association for Endoscopic Surgery concluded that laparoscopy can be used for incarcerated inguinal hernias, especially the TAPP regimen (17).

3. Skilled endoscopic techniques can ensure the therapeutic effect and reduce hospitalization costs.

Although robot assistance was not used, our laparoscopic surgical results were comparable to those of robotic surgery in high-income countries. Much of the research on robot-assisted emergency hernia surgery has sidestepped economic concerns (18, 19). This highlights the understated fact that standard laparoscopy, when performed by a skilled surgeon, reduces the cost of machinery while guaranteeing therapeutic efficacy. We chose laparoscopic surgery for incarcerated hernia, which was more cost-effective than open surgery (20). For patients facing the double burden of increasing hernia prevalence and financial hardship, our study protocol provides good ideas for sustainable quality improvement, a perspective that has been seriously neglected in current research-focused Western research protocols, while also meeting the healthcare reform measures proposed in our country. In addition, a longer length of stay is associated with increased medical costs and resource utilization. By optimizing surgical techniques and improving management practices, hospitals can reduce the length of stay, resulting in substantial cost savings. ERAS programs, like the one we joined, not only improve patient outcomes, but also improve the efficiency of medical services, which is a new idea worth recommending.

## Conclusion

Laparoscopic surgery combined with the ERAS protocol can safely re-consider a new concept for the treatment of incarcerated inguinal hernia. Moreover, compared with open surgery, it can significantly shorten the hospital stay and reduce the incidence of complications. Despite limitations such as single-center design and lack of long-term follow-up data, this cost-effective model advances accurate emergency surgery.

## Data Availability

The datasets presented in this study can be found in online repositories. The names of the repository/repositories and accession number(s) can be found in the article/Supplementary Material.
